# Investigating the prediction of CpG methylation levels from SNP genotype data to help elucidate relationships between methylation, gene expression and complex traits

**DOI:** 10.1002/gepi.22496

**Published:** 2022-08-05

**Authors:** James J. Fryett, Andrew P. Morris, Heather J. Cordell

**Affiliations:** ^1^ Population Health Sciences Institute, Faculty of Medical Sciences Newcastle University Newcastle upon Tyne UK; ^2^ Centre for Genetics and Genomics Versus Arthritis, Centre for Musculoskeletal Research University of Manchester Manchester UK

**Keywords:** MetaXcan, MWAS, PrediXcan, TWAS

## Abstract

As popularised by PrediXcan (and related methods), transcriptome‐wide association studies (TWAS), in which gene expression is imputed from single‐nucleotide polymorphism (SNP) genotypes and tested for association with a phenotype, are a popular approach for investigating the role of gene expression in complex traits. Like gene expression, DNA methylation is an important biological process and, being under genetic regulation, may be imputable from SNP genotypes. Here, we investigate prediction of CpG methylation levels from SNP genotype data to help elucidate relationships between methylation, gene expression and complex traits. We start by examining how well CpG methylation can be predicted from SNP genotypes, comparing three penalised regression approaches and examining whether changing the window size improves prediction accuracy. Although methylation at most CpG sites cannot be accurately predicted from SNP genotypes, for a subset it can be predicted well. We next apply our methylation prediction models (trained using the optimal method and window size) to carry out a methylome‐wide association study (MWAS) of primary biliary cholangitis. We intersect the regions identified via MWAS with those identified via TWAS, providing insight into the interplay between CpG methylation, gene expression and disease status. We conclude that MWAS has the potential to improve understanding of biological mechanisms in complex traits.

## 1 INTRODUCTION

Genome‐wide association studies (GWAS) have successfully identified regions of the genome associated with a range of phenotypes (MacArthur et al., 2017). However, for many of these findings, the mechanism by which variants affect their associated phenotype remains unknown (Gallagher & Chen‐Plotkin, 2018). Most trait‐associated variants identified by GWAS fall in regulatory regions of the genome (Maurano et al., 2012), and are hypothesised to act by altering gene expression rather than the protein code. Indeed, enrichment of expression quantitative trait loci (eQTLs) at known GWAS risk loci (Nicolae et al., 2010), and overlaps between GWAS risk variants and genomic loci affecting markers of genome regulation (such as histone modifications) have been identified (Chen et al., 2016; Tehranchi et al., 2016; X. Zhang, Joehanes, et al., 2015), reinforcing this hypothesis. For this reason, an approach to improve understanding of mechanisms underlying GWAS findings is to integrate GWAS and gene expression data. One such approach is the transcriptome‐wide association study (TWAS), implemented in the software packages PrediXcan (Gamazon et al., 2015), S‐PrediXcan (Barbeira et al., 2018) and FUSION (Gusev et al., 2016). This approach uses known relationships between single‐nucleotide polymorphisms (SNPs) and gene expression (estimated from a reference panel with matched genotype and gene expression data) to impute expression into GWAS samples. Imputed expression is then tested for association with the phenotype to identify phenotype‐relevant genes. This method has been widely used to investigate the role of gene expression in complex traits (Ioannidis et al., 2018; Khawaja et al., 2018; Mancuso et al., 2018; Roselli et al., 2018), and represents a powerful approach for interpretation of GWAS findings.

CpG methylation, which refers to the addition of a methyl (–CH3) group to cytosine residues in cytosine‐guanine dinucleotides, is known to regulate the expression of nearby genes. For example, increased methylation at CpG sites in promoter regions is often associated with decreased expression at a nearby gene (although the relationship is often more complex than this) (Luo et al., 2018; Schubeler, 2015). Additionally, aberrant methylation at CpG sites has been implicated as a potential mechanism in complex diseases (Dhana et al., 2018; Story Jovanova et al., 2018; Xu et al., 2018). Like gene expression, DNA methylation is under genetic regulation. Twin and family‐based studies have identified a significant heritable component of CpG methylation, with estimates of heritability ranging from 16% to 20% (Bell et al., 2012; Grundberg et al., 2013; Hannon, Knox, et al., 2018; van Dongen et al., 2016). Studies estimating CpG methylation heritability using SNPs also find a significant heritable component, although estimates vary depending on which SNPs are used. For example, a large study using SNPs across the whole genome found an estimate of 19% (van Dongen et al., 2016), similar to estimates from twin studies, whereas studies focussing on heritability attributable to SNPs proximal to CpG sites generated smaller estimates (Quon et al., 2013; Rowlatt et al., 2016). In addition, studies have consistently identified relationships between CpG methylation and genotypes at individual SNPs, termed methylation quantitative trait loci (mQTLs) (Gaunt et al., 2016; Grundberg et al., 2013; Richardson et al., 2016; Volkov et al., 2016). The presence of these mQTLs and the non‐zero heritability estimates of CpG methylation indicate that it may be possible to predict methylation from SNP genotypes.

Here, we apply the PrediXcan approach, originally designed for testing for association between predicted gene expression levels and a phenotype, to the problem of testing for association between predicted methylation levels and a phenotype, assuming that genome‐wide SNP data is available (in both training and test data sets) to inform the prediction. We start by investigating how well CpG methylation can be predicted from SNP genotypes local to CpG sites, using data from the Accessible Resource for Integrated Epigenomics Studies (ARIES) (Relton et al., 2015), a study within the Avon Longitudinal Study of Parents and Children (ALSPAC) (Boyd et al., 2013; Fraser et al., 2013) and data from the Understanding Society study (Hannon, Gorrie‐Stone, et al., 2018). We compare the performance of three penalised regression methods and investigate prediction accuracy at five window sizes to identify an optimal method and window size for prediction model training. For CpG sites where methylation can be predicted well, we then generate prediction models and illustrate their use in a methylome‐wide association study (MWAS) of the autoimmune liver disease primary biliary cholangitis (PBC). Finally, we investigate the relationships between regions identified via MWAS and those identified via TWAS, providing insight into the interplay between CpG methylation, gene expression and disease status.

## 2 MATERIALS AND METHODS

### 2.1 ARIES data

We obtained approval to access genotype data and CpG methylation data measured at an antenatal clinic for 855 mothers as part of the ARIES study, a study within ALSPAC (Boyd et al., 2013; Fraser et al., 2013). The ALSPAC study website contains details of all the data that is available through a fully searchable data dictionary and variable search tool (http://www.bristol.ac.uk/alspac/researchers/our-data/). Ethical approval for the study was obtained from the ALSPAC Ethics and Law Committee and the Local Research Ethics Committees. Informed consent for the use of data collected via questionnaires and clinics was obtained from participants following the recommendations of the ALSPAC Ethics and Law Committee at the time. Consent for biological samples has been collected in accordance with the Human Tissue Act (2004).

Details of the collection and processing of the ARIES data can be found in the Supporting Information Text. Following processing and quality control, we were left with matched genotype and CpG methylation data at the “antenatal” time point for 841 ARIES samples to be taken forward for statistical modelling.

### 2.2 Understanding Society data

We also obtained approval to access genotype data (University of Essex et al., 2015) and methylation data (University of Essex et al., 2017) previously generated by Understanding Society: the UK Household Longitudinal Study. Details of the collection and processing of the Understanding Society data can be found in the Supporting Information Text. Following processing and quality control, we had matched genotype and methylation data for 1120 samples to be taken forward for downstream analysis.

### 2.3 Training and testing CpG methylation prediction models

CpG methylation prediction models were generated separately in the ARIES and Understanding Society data sets by regressing methylation levels on genotype dosages of all SNPs within a specified distance (window size) of the CpG site, using three different penalised regression methods (see Supporting Information Text). The methods considered were ridge regression (Hoerl & Kennard, 2000), LASSO (Tibshirani, 1996) and elastic net with mixing parameter α set to 0.5 (Zou & Hastie, 2005). Although, in principle, one could consider the mixing parameter α as a parameter to be estimated (e.g., by performing a grid search), fixing its value at 0.5 has the advantage of reducing the computational complexity and matches what was done in the original PrediXcan publication (Gamazon et al., 2015), effectively providing a balance between the level of penalisation employed by ridge regression and LASSO. The prediction models were trained in R using the *glmnet* package (Friedman et al., 2010). For all methods, a value for the regularisation parameter *λ* was selected using 10‐fold cross‐validation. Any values of *λ* that produced a prediction model that did not contain any SNPs were excluded. Of the remaining values of *λ*, the value at which the minimum mean squared error between predicted and observed methylation was achieved in the cross‐validation was then selected.

The ARIES data were used to carry out a comparison of penalised regression approaches, while both ARIES and Understanding Society data were used to carry out a comparison of SNP window sizes. To compare the three penalised regression approaches, 50% of ARIES samples were designated as the model training set, and 20% as the test set. (The remaining 30% of samples were saved for use later as a prediction model testing set). For each CpG site, prediction models were trained by regressing CpG methylation on SNP genotypes for all SNPs within 1 Mb of the CpG site using each method, the resulting models were applied to the test set, and the correlation (*R*) between predicted and observed methylation levels was calculated.

For the comparison of five window sizes, CpG methylation prediction models were trained by regressing CpG methylation on genotypes of SNPs within 250 kb, 500 kb, 1 Mb, 2 Mb or 3 Mb of the CpG site, using elastic net with *α* = 0.5 and using the same training and testing sets as used previously to compare penalised regression methods. By limiting the maximum window size to 3MB, we effectively focus on SNPs that act as *cis* mQTLs; although a role has been demonstrated for *trans* mQTLs (including both interchromosomal effects and intra‐chromosomal effects, operating at distances >5 Mb of the CpG site) (Min et al., 2020), they represent a much smaller percentage of total mQTLs (8.5% compared to the 92.5% represented by *cis* mQTLs) and, moreover, the effect sizes for *trans* mQTLs are lower than for *cis* mQTLs, meaning that much larger sample sizes are required to reliably identify them (Min et al., 2020).

Having identified an optimal method from the three methods considered, and a CpG‐specific window size for prediction model training, new CpG methylation prediction models were trained using the ARIES and Understanding Society data sets, to establish how accurately methylation could be predicted. The 50% of ARIES samples that had previously been used as a prediction model training set and the 20% that had previously been used for prediction model testing were combined and used as the prediction model training set here. The remaining 30% of samples that had not been used before this point were used as a prediction model testing set to evaluate overall predictive accuracy. The same procedure was used to generate training and testing sets for assessing predictive accuracy with the Understanding Society data.

### 2.4 Enrichment testing

Having estimated predictive accuracy, enrichment tests were performed to determine the extent to which five pre‐specified functional annotations were more highly represented among the set of well‐predicted CpG sites than among the background set of all CpG sites that passed quality control. Separately, we also tested whether the same five annotations were more highly represented among the set of trait‐associated CpG sites than among all CpG sites tested in the MWAS. Enrichment tests were applied to the results obtained using ARIES data and Understanding Society data using annotations taken from the Illumina manifest files. As the annotations listed in the 450k chip and EPIC chip manifest files were slightly different, separate enrichment tests were performed for the results obtained using ARIES data and Understanding Society data. For full details of the procedure, see the Supporting Information Text.

### 2.5 Heritability estimation

The heritability of methylation at each CpG site was estimated using restricted maximum likelihood (REML) analysis in GCTA (Yang et al., 2010, 2011). Heritability estimates were generated separately for the ARIES data and the Understanding Society data. For each CpG, the SNPs within the optimal window size of the CpG site's genomic location were used to construct a genetic relationship matrix (GRM). The proportion of the variance of CpG methylation explained by these SNPs (the narrow‐sense heritability) was then estimated using REML analysis in GCTA. Heritability estimates were restricted to fall within the [0, 1] range.

### 2.6 Training and validating a final set of CpG methylation prediction models

Having obtained an estimate of prediction accuracy using the optimal method and window size for each CpG site, our final step was to train a set of CpG methylation prediction models that could be used in MWAS to investigate the relationship between predicted CpG methylation and complex traits.

To maximise the prediction accuracy of these final CpG methylation prediction models (and thus improve the power of the subsequent tests of association between predicted methylation and phenotype), the sample size of the prediction model training set was increased by combining the 70% training set and 30% testing set. This resulted in two training sets (one comprised of ARIES data and one of Understanding Society data), each consisting of 100% of their samples. Using these training sets, prediction models were then trained for the CpG sites where a prediction accuracy estimate ≥ 0.1 had been achieved at the optimal method and window size. This resulted in 78,250 CpG methylation prediction models trained using 100% of the ARIES data and 207,525 CpG methylation prediction models trained using 100% of the Understanding Society data.

The prediction models were then validated through application to the other data set on which the models had not been trained (see Supporting Information Text). The prediction models trained on 100% of ARIES data were applied to 100% of the Understanding Society samples, and the correlation between predicted and observed methylation was calculated. Similarly, prediction models trained on 100% of Understanding Society data were applied to 100% of the ARIES samples, and the correlation between predicted and observed methylation was calculated. Models which failed to meet a prediction accuracy *R* estimate ≥ 0.1 in their respective validation data set were discarded from further consideration.

### 2.7 MWAS of PBC

As an illustration of our approach, the final CpG methylation prediction models were used within the S‐PrediXcan (Barbeira et al., 2018) software package, together with summary statistics from our recent genome‐wide meta‐analysis of the autoimmune liver disease PBC (Cordell et al., 2021), to perform an MWAS, testing for association between predicted methylation at up to 78,250 ARIES and 207,525 Understanding Society CpGs and disease status. This analysis represents an updated version of the analysis previously described (Cordell et al., 2021) which had used earlier, unoptimised, versions of the methylation prediction models. We also used the most recent (GTEx v8) gene expression and splicing (eQTL and sQTL) prediction models from the PredictDB Data Repository (https://predictdb.org/) in S‐PrediXcan, together with the same set of PBC summary statistics, to test for association between disease status and predicted gene expression/splicing, allowing us to intersect the regions identified via MWAS with those identified via TWAS and a splicing site wide association study (SWAS), respectively.

## 3 RESULTS

### 3.1 Sparse methods outperform polygenic methods at prediction of CpG methylation from SNP genotypes

**Figure 1:**
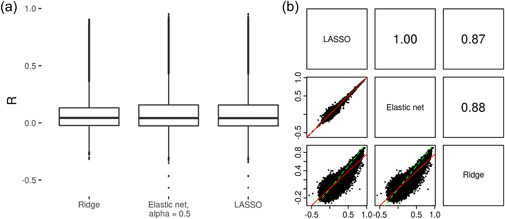
Comparison of penalised regression approaches for predicting CpG methylation. (a) Box plots of prediction accuracy estimates (*R*) from training and testing prediction models using 3 forms of penalised regression (ridge regression, elastic net, LASSO) on ARIES data. The line within the box represents the median, with the edges of the box the upper and lower quartiles. (b) Correlation plots between prediction accuracy estimates achieved using the three penalised regression approaches. In the lower panels, each point represents a CpG site, with the *R* achieved by two methods displayed on the axes. Also shown are the line of equality (green dashed line) and a best fit line between *x* and *y* (red solid line). Upper panels show the pairwise correlations between the *R* values achieved using the three methods.

We first compared the performance of three penalised regression approaches for the prediction of CpG methylation from local SNP genotypes. Overall, sparse approaches (elastic net and LASSO) outperformed the more polygenic ridge regression approach (Figure 1a). Across the CpG sites successfully modelled with all three methods, higher average prediction accuracy estimates were achieved with LASSO (mean *R* = 0.097, SD = 0.191) and elastic net (mean *R* = 0.098, SD = 0.191) than with ridge regression (mean *R* = 0.080, SD = 0.164). Estimates from all three methods were highly correlated (Figure 1b). While the difference between methods was hard to see when considering all CpGs, it could be seen more clearly when looking at the 10,004 CpGs for which an *R* estimate ≥ 0.5 was achieved by any of the three methods (Supporting Information: Figure S1).

### 3.2 The optimal window size for fitting CpG methylation prediction models is CpG‐specific

**Figure 2:**
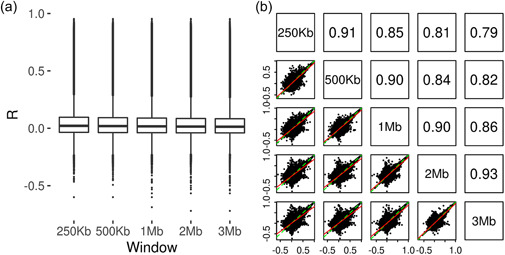
Comparison of window sizes for predicting CpG methylation. (a) Box plots of prediction accuracy estimates (*R*) from training and testing prediction models using elastic net with single‐nucleotide polymorphisms selected using five window sizes (250 kb, 500 kb, 1 Mb, 2 Mb and 3 Mb) on ARIES data. The line within the box represents the median, with the edges of the box the upper and lower quartiles. (b) Correlation plots between prediction accuracy estimates achieved using the five window sizes. In the lower panels, each point represents a CpG site, with the *R* achieved at the two window sizes displayed on the axes. Also shown are the line of equality (green dashed line) and a best fit line between *x* and *y* (red solid line). Upper panels show the pairwise correlations between the *R* values achieved at the five window sizes.

We next sought to investigate whether changing the window size used to select SNPs used for model fitting could improve the models’ accuracy. Prediction accuracy estimates obtained at the five window sizes were highly correlated with one another (Figure 2a,b). On average, a slight decrease in the mean accuracy achieved across the CpG sites successfully modelled at all five window sizes was observed with an increase in the window size (Table 1). However, when looking at the results on a CpG‐by‐CpG basis, no clear pattern was observed, with some CpG sites showing greater prediction accuracy at the larger window sizes and other CpG sites showing greater prediction accuracy at the smaller window sizes. The same result was observed when restricting the comparison to those CpG sites for which an *R* estimate ≥ 0.5 was achieved at any of the five window sizes (Supporting Information: Figure S2). This suggests that the optimal window size for training CpG methylation prediction models is a CpG‐specific quantity. For each CpG site, the optimal window size was therefore determined as the window size at which the maximum prediction accuracy was achieved.

**Table 1:** Average prediction accuracy estimates achieved when training and testing CpG methylation prediction models using five different window sizes using ARIES data.

Window size	Average prediction accuracy
250 kb	0.0548
500 kb	0.0525
1 Mb	0.0502
2 Mb	0.0481
3 Mb	0.0467

The same comparison of five window sizes was performed on the other data set considered, the Understanding Society study, to identify optimal window sizes for those CpG methylation measurements. 50% of Understanding Society samples were designated as the training set, with 20% of samples assigned to the testing set. Overall, prediction accuracy estimates at the five window sizes were highly correlated with one another (Supporting Information: Figure S3). Again, some CpG sites showed greater prediction at larger window sizes, while others showed greater accuracy at the smaller window sizes, reinforcing the conclusion that the optimal window size for CpG methylation prediction model training is CpG‐specific. The same conclusion was once again reached when the comparison was restricted to just the CpG sites where a prediction accuracy estimate ≥ 0.5 was achieved with any of the five window sizes (Supporting Information: Figure S4). For each CpG site in the Understanding Society data set, the optimal window size was therefore determined as the window size at which the maximum prediction accuracy was achieved.

### 3.3 Methylation at most CpG sites cannot be accurately predicted from SNP genotypes

Having identified an optimal method (elastic net with α set to 0.5) from the three methods considered, and a CpG‐specific window size for prediction model training, new CpG methylation prediction models were trained in the 70% of the ARIES and Understanding Society data sets using these optimal values. The prediction models were then applied to their respective 30% testing sets (i.e., ARIES‐trained models applied to the ARIES testing set and Understanding Society‐trained models applied to the Understanding Society testing set), and the correlation between predicted and measured methylation was calculated.

**Figure 3:**
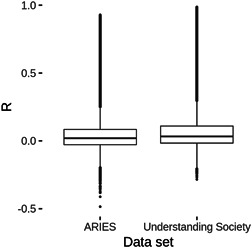
Prediction accuracy of CpG methylation prediction models trained using elastic net with *α* = 0.5 and with a CpG‐specific window size. Box plots of prediction accuracy estimates (*R*) from training and testing prediction models using elastic net (with *α* = 0.5) with a CpG‐specific window size using data from ARIES and Understanding Society. The line within the box represents the median, with the edges of the box the upper and lower quartiles.

**Figure 4:**
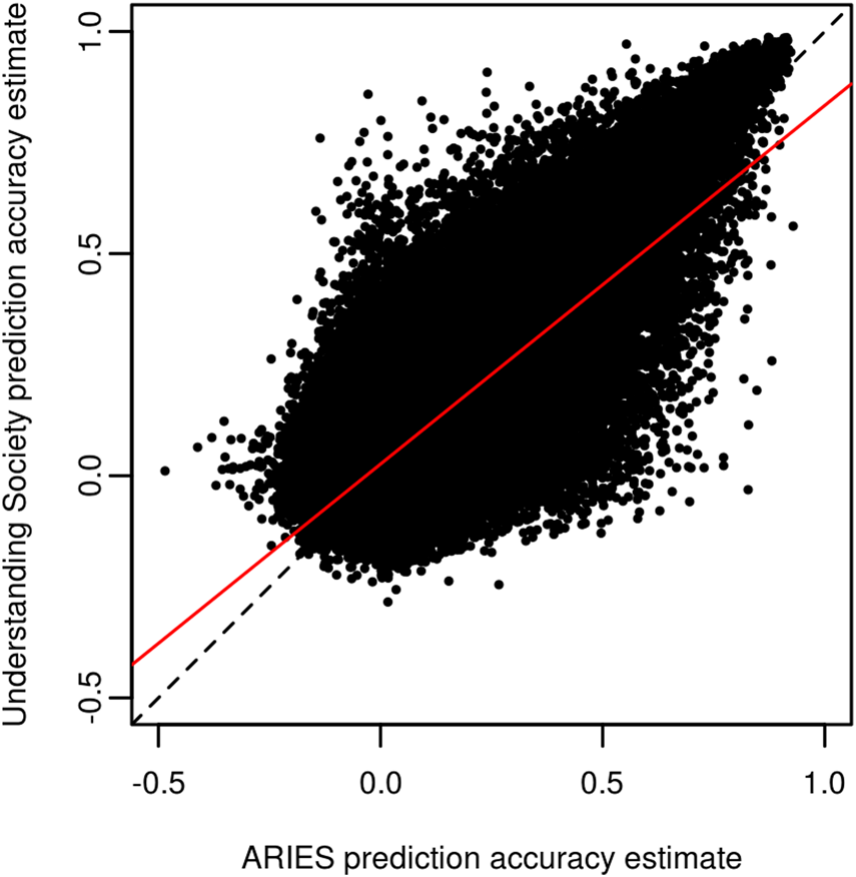
Comparison of prediction accuracy estimates from ARIES and Understanding Society data sets. Each point represents a CpG site, with its prediction accuracy estimate obtained from training and testing a prediction model using the ARIES data shown on the *x* axis, and its prediction accuracy estimate obtained from training and testing a prediction model using the Understanding Society data shown on the *y* axis. The red line represents a best fit line, and the dashed line represents the line of equality (*y* = *x*).

We found that methylation at most CpG sites could not be accurately predicted from SNP genotypes (Figure 3), although there existed a subset of CpG sites for which methylation could be predicted with some accuracy. Reassuringly, prediction accuracy estimates from ARIES data were highly correlated (*r* = 0.757) with those obtained from Understanding Society data (Figure 4). This was especially the case for the well‐predicted CpG sites such as cg16906346, which showed a prediction accuracy of 0.924 when examined using ARIES data and a prediction accuracy estimate of 0.953 when examined using Understanding Society data. In total, prediction models for 78,250 CpG sites from ARIES (assayed using the HumanMethylation450 BeadChip) and 207,525 CpG sites from Understanding Society (assayed using the denser MethylationEPIC array) showed a prediction accuracy ≥ 0.1 when predicted using the optimal method and window size. These CpG sites were taken forward for further analysis.

Of particular interest were the CpG sites where methylation could be predicted with a high degree of accuracy. 10,220 ARIES‐trained models and 30,865 Understanding Society‐trained models showed prediction accuracy ≥ 0.5 in their respective test sets, representing a set of well‐predicted CpG sites. To learn more about these well‐predicted CpG sites, enrichment testing was conducted. When considering the CpGs that were predicted well when using ARIES data, CpG sites tagged to genes (odds ratio [OR] = 0.682, *p* = 4.08 × 10^−68^), CpG sites located at CpG islands (OR = 0.883, *p* = 1.72 × 10^−9^) and CpG sites tagged to promoters (OR = 0.636, *p* = 1.13 × 10^−61^) were all depleted among the set of well‐predicted CpG sites (when compared to the background set of all CpG sites), while CpGs at enhancer regions (OR = 1.42, *p* = 9.62 × 10^−53^) and CpGs at DNAse1 hypersensitivity sites (OR = 1.40, *p* = 8.57 × 10^−33^) were enriched among the well‐predicted CpG sites. Reassuringly, these enrichments and depletions were replicated when looking at those CpG sites that were well‐predicted when using the Understanding Society data (Supporting Information: Figure S5).

The theoretical upper limit on how accurately CpG methylation can be predicted from SNP genotypes is equivalent to its narrow‐sense heritability. We estimated the heritability of methylation at each CpG site using SNPs within the CpG‐specific optimal window size and compared heritability estimates with estimates of prediction accuracy obtained with the optimal method and window size. Overall, heritability estimates were highly correlated and concordant with prediction accuracy estimates, with the exception of a small number of CpG sites where heritability estimates were much greater than the prediction accuracy estimates). This was observed for both the ARIES and the Understanding Society data sets (Supporting Information: Figure S6), suggesting that the upper bound on prediction accuracy had been reached for most CpG sites.

### 3.4 Training and validating a final set of CpG methylation prediction models

Having obtained an estimate of prediction accuracy using the optimal method and window size for each CpG site, the final step was to use the full set (100%) of samples in the ARIES and Understanding Society data sets to train and validate a set of CpG methylation prediction models that could be used in MWAS to investigate the relationship between predicted CpG methylation and complex traits. Following this procedure (see Supplementary Text for full details), we ended up with a total of 232,356 prediction models. These 232,356 prediction models covered 193,315 unique CpG sites, with 39,041 CpG sites represented by both an ARIES‐trained and an Understanding Society‐trained prediction model.

### 3.5 MWAS in PBC identifies regions that overlap with TWAS and SWAS signals

**Figure 5:**
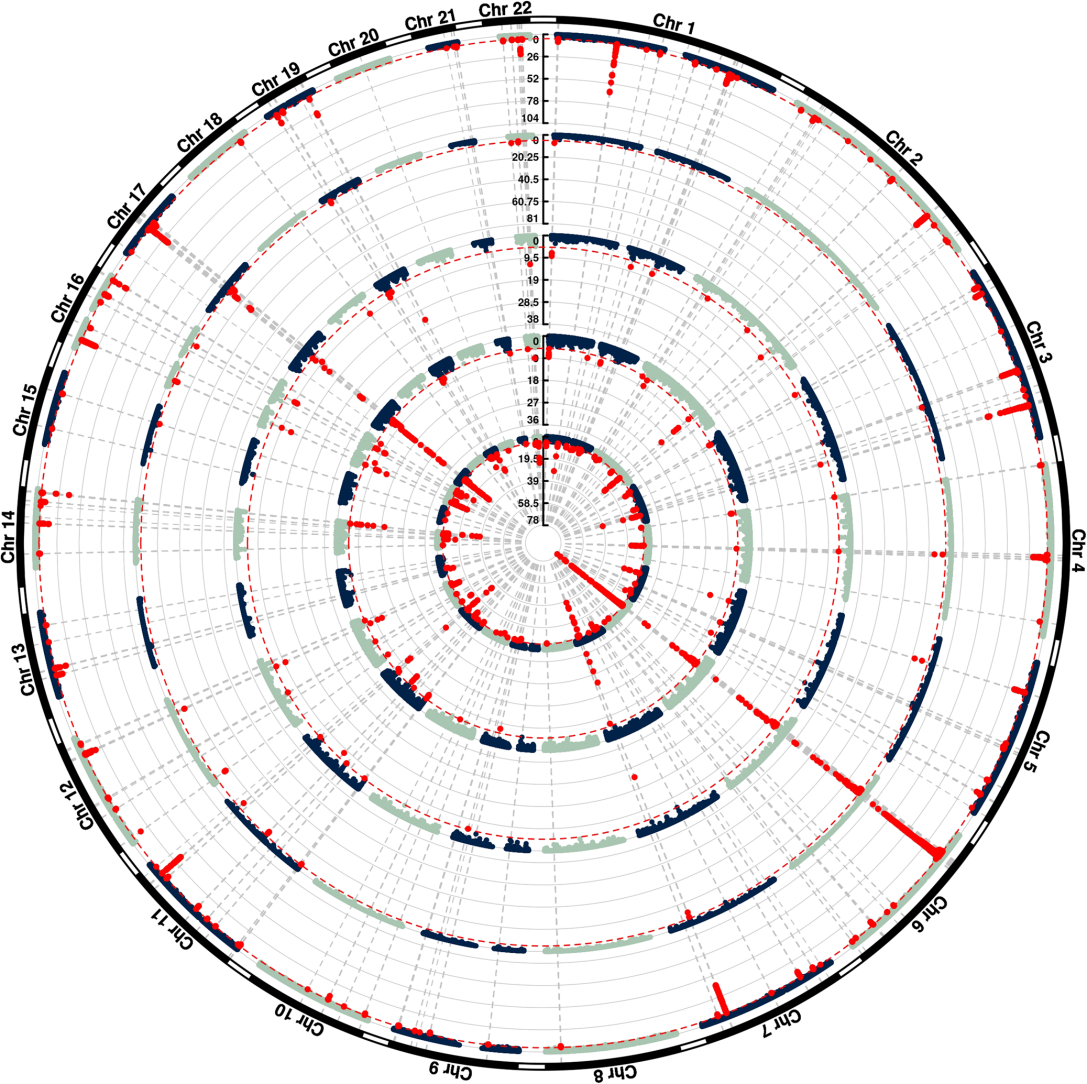
Circular Manhattan plot. Shown are the −log10 *p* values from tests of association with PBC and predicted methylation at Understanding Society CpGs (outermost circle), predicted methylation at ARIES CpGs (second circle), predicted gene expression (third circle), predicted splicing (fourth circle) and measured (genotyped or imputed) SNPs (innermost circle). PBC, primary biliary cholangitis; SNP, single‐nucleotide polymorphism.

**Figure 6:**
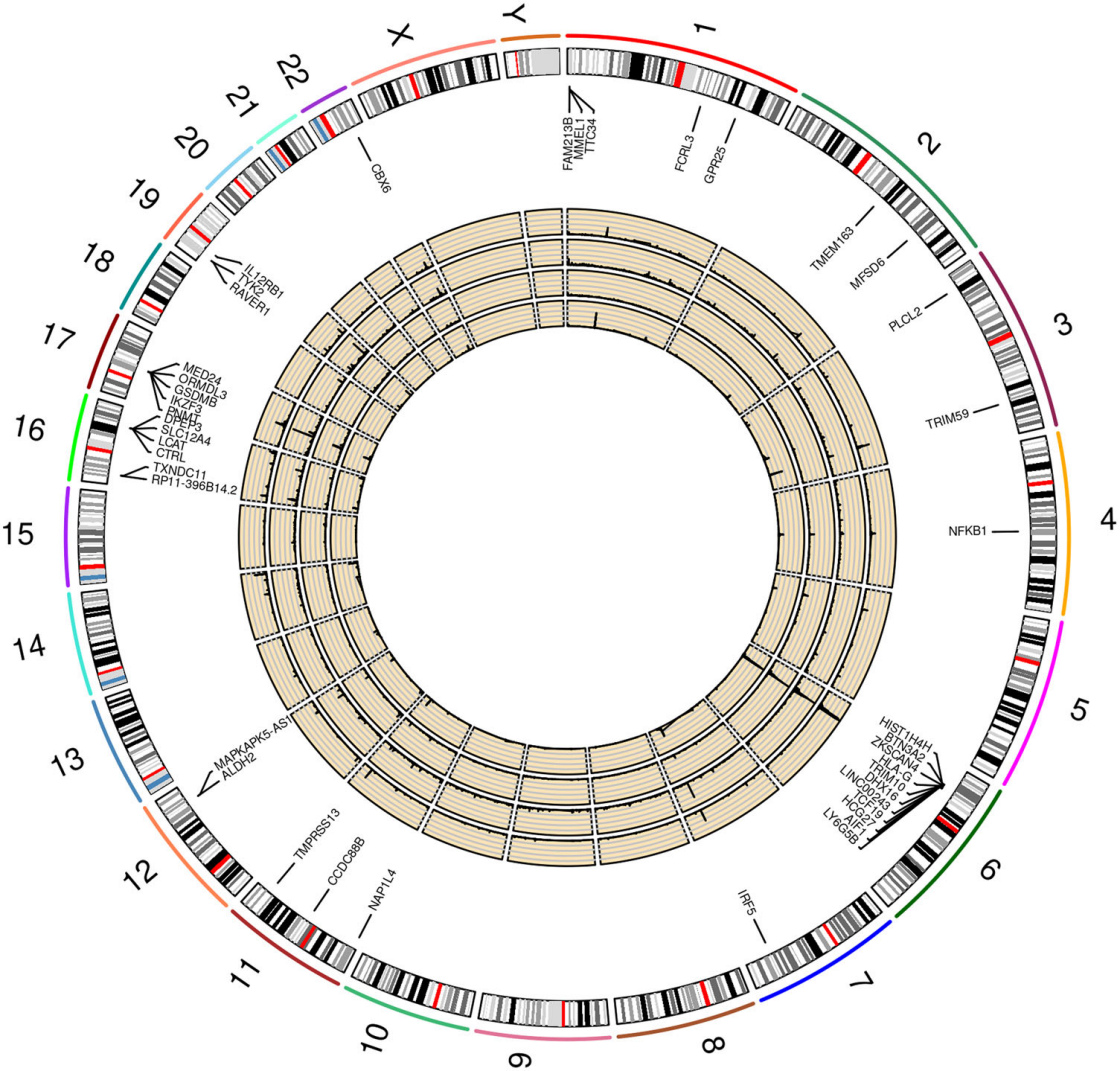
Circos plot. Shown via links to the ideogram are the significant genes along with inner circles showing the −log10 *p* values from tests of association with PBC and predicted methylation at Understanding Society CpGs (outermost circle), predicted gene expression (second circle), predicted splicing (third circle) and measured (genotyped or imputed) SNPs (innermost circle). PBC, primary biliary cholangitis; SNP, single‐nucleotide polymorphism.

MWAS was carried out to test for association between predicted methylation and disease status at the 48,658 (out of a possible 78,250) ARIES CpGs and 172,008 (out of a possible 207,525) Understanding Society CpGs for which sufficient SNPs were available in the PBC summary statistics to inform the tests. Results are shown in Figure 5, along with TWAS and SWAS results from similar tests of association between PBC and predicted gene expression and splicing, respectively. The association results generated at the Understanding Society CpGs (outermost circle) were considerably stronger than those generated at the sparser set of ARIES CpGs (second circle), resulting in 782 significant Understanding Society CPGs (p < 2.91 × 10^−7^, corresponding to *p* = 0.05 Bonferroni‐corrected for the 172,008 tests performed). As expected, these significant Understanding Society CpGs corresponded to GWAS association signals of association between SNPs and PBC (innermost circle), but also, in many cases, to TWAS and SWAS signals (i.e., regions of association between PBC and predicted gene expression [third circle] and/or splicing [fourth circle]). Figure 6 shows the implicated genes (*p* < 7.79 × 10^−6^, corresponding to *p* = 0.05 Bonferroni‐corrected for the 6419 genes tested)—except for some regions on chromosomes 6 and 17 where there were too many significant genes to be plotted—along with MWAS results at Understanding Society CpGs (outermost circle), TWAS (second circle) results, SWAS results (third circle) and GWAS results (innermost circle). A full list of the 63 implicated genes is given in Supporting Information: Table 1, while Supporting Information: Table 2 additionally includes the significant CPGs and significant splicing sites (*p* < 6.39 × 10^−6^, corresponding to *p* = 0.05 Bonferroni‐corrected for the 7825 tests performed). Further detailed analysis of each of the 63 implicated gene regions, using fine‐mapping and complementary approaches such as co‐localisation (Giambartolomei et al., 2018), Mendelian Randomisation (Zuber et al., 2022), and Bayesian Network analysis (Howey et al., 2020, 2021) is beyond the scope of the current study, but will be carried out in the near future to help elucidate the underlying causal relationships between the different biological phenomena investigated here and the extent to which the same genetic factor(s) may underpin the associations observed.

## 4 DISCUSSION

Here we have extended the approach originally popularised by PrediXcan (Gamazon et al., 2015) for testing association between predicted gene expression levels and a phenotype, to instead test for association between predicted *methylation* levels and a phenotype, allowing one to carry out MWAS to identify associations between the trait and imputed methylation at CpGs across the genome. In the original PrediXcan publication (Gamazon et al., 2015), the authors considered prediction models based on LASSO, elastic net with *α* = 0.5 and polygenic scores. They found that LASSO performed similarly to elastic net and both methods outperformed polygenic scores; they subsequently focused on prediction models using elastic net because it performed well and was more robust to slight changes in input SNPs.

Comparing the performance of penalised regression approaches for predicting methylation, similarly to what we had seen previously for gene expression (Fryett et al., 2020), we found that sparse models (trained with LASSO or elastic net with *α* = 0.5) tended to outperform more polygenic models (trained with ridge regression), suggesting that the underlying local genetic architecture of CpG methylation is sparse. While there has been no formal investigation of the genetic architecture of CpG methylation, mQTL studies have found that most CpG sites have few mQTLs (if any), each with a large effect size (Gaunt et al., 2016), which is indicative of a sparse local architecture. Gene expression has also been shown to have a sparse local architecture (Wheeler et al., 2016), indicating this local sparsity may a feature shared by multiple cellular traits.

A comparison of prediction accuracy estimates when training CpG methylation prediction models using a range of different window sizes showed that increasing window size led to a marginal decrease on average prediction accuracy across many CpG sites, although the effect on the prediction accuracy of individual CpG sites varied. Interestingly, there was no consistent direction of effect to this, with some CpG sites benefitting from a smaller window size, and others benefitting from a larger window size, suggesting that the optimal window size for the prediction of CpG methylation is a CpG‐specific quantity. Before our analysis, there had been no investigation into the effect of window size on the accuracy with which CpG methylation can be predicted from SNP genotypes. However, given that mQTL studies have shown that methylation at a small number of CpGs is regulated by SNPs distal to the CpG sites (Gaunt et al., 2016), it is perhaps unsurprising that increasing the window size to the point where some of these more distal regulatory SNPs can be included in the prediction models could improve prediction accuracy for some CpG sites. In contrast, increasing the window size for the CpG sites where methylation is not known to be regulated by distal SNPs could lead to increased noise in the CpG methylation prediction model fitting procedure, leading to poorer estimation of the prediction model coefficients, and subsequently poorer prediction accuracy. Given that these distal regulatory SNPs are only known to exist for some, not all, CpG sites, this may explain why the average prediction accuracy across all CpG sites examined here fell slightly as the window size increased.

A crucial finding from our study is that methylation at most CpG sites cannot be accurately predicted from SNP genotypes. Through comparison with heritability estimates obtained using GCTA, we found that prediction accuracy estimates for most CpG sites examined here approached their upper bound, and so are unlikely to be increased much further at the current window size and sample size. Despite most CpG sites showing poor prediction accuracy and heritability estimates, there exist a set of CpG sites where methylation can be predicted with accuracy, with some CpG sites showing high degrees of prediction accuracy. We found that these well‐predicted CpG sites are enriched at enhancers and depleted at promoters, matching previously identified enrichments for CpG sites with mQTLs (Banovich et al., 2014; Gutierrez‐Arcelus et al., 2013). Importantly, prediction accuracy estimates for well‐predicted CpG sites replicated when tested on an independent data set, indicating that we have identified real, reliable relationships between genotype and measured methylation. For these robustly predicted CpG sites, our approach represents a powerful method to investigate their role in complex traits.

As an illustration of our approach, we applied our final CpG methylation prediction models to GWAS summary data from PBC, identifying 782 significant CpGs, many of which localised with significant regions of association between predicted gene expression and/or splicing. Further interrogation of these regions using advanced co‐localisation and causal modelling analysis techniques will help elucidate the interplay between CpG methylation, gene expression and disease status, thus improving our understanding of underlying biological mechanisms. This PBC data set was chosen largely for convenience (as we had easy access to the GWAS summary statistics, generated previously by ourselves) but also partly because our previous work with PBC led us to expect the existence of strong GWAS signals, making this a good data set to illustrate the approach; in principle any disease exhibiting similarly strong GWAS signals should work equally well.

We conclude by briefly discussing some limitations of our study. We trained CpG methylation prediction models using methylation data measured in blood, since the only large‐scale data sets available with both genome‐wide SNP data and genome‐wide methylation are blood‐based. However, blood is unlikely to be the true causal tissue of interest for many complex traits. Studies have found strong concordance between the effects of SNPs on methylation in blood and the effects of those same SNPs on methylation in other tissues (Hannon et al., 2017; Lin et al., 2018; Shi et al., 2014), however, the number of tissues studied and the sample sizes used in these studies has been limited. Thus, it is difficult to say how well our prediction models or association results translate to tissues of interest. Additionally, both data sets used to generate prediction models are from populations of British ancestry. To date, there has been little study of how genetic effects on methylation differ across populations. In TWAS, gene expression prediction accuracy is reduced when using prediction models trained using samples of a different ancestry to the samples in the GWAS data (Mikhaylova & Thornton, 2019; Mogil et al., 2018). Should the CpG methylation prediction models generated here be used in an MWAS of a non‐European population, a similar reduction in prediction accuracy would likely be observed.

In our study, we focussed on comparing a limited set of (penalised regression based) prediction methods and a limited set of five possible window sizes for choosing the SNP predictors. This was in part motivated by the success of such approaches for predicting gene expression (Gamazon et al., 2015) for use in subsequent association testing (via TWAS) with a phenotype of interest. Although alternative methods (e.g., based on support vector machines or deep learning) have been developed to predict DNA methylation (Bhasin et al., 2005; Levy et al., 2020; Tang et al., 2020; Tian et al., 2019; W. Zhang, Spector, et al., 2015; Zhou et al., 2012), these methods are not generally designed to predict methylation from SNP genotype data alone (as would be needed to take the models forward for MWAS, in conjunction with individual‐level SNP genotypes or GWAS summary statistics for a phenotype of interest), but they rather make use of additional features (such as full DNA sequence data, histone modification marks or transcription factor binding sites) to inform the prediction. This is a much richer set of features than would generally be available in publicly (or privately) available GWAS data sets, limiting the applicability for subsequent MWAS of any models that encompass these features.

Limiting the search for SNP predictors to five possible (CpG‐specific) window sizes was largely a pragmatic choice. It is possible that improved prediction for any given CpG could be achieved through use of a window size not considered here, or through a more complicated scheme such as adapting the window size to the local LD pattern. However, we note that optimal prediction of methylation per se is not our ultimate goal; we are more interested in the power to detect associations through MWAS, which relies not only on the accuracy of the methylation imputation, but also on the sample size of the GWAS data (or summary statistics) to be used. Thus, an association can still be detected for CpGs with low prediction R, provided a GWAS with a sufficiently large sample size is used.

In conclusion, our study suggests that MWAS based on imputed methylation levels represents a potentially powerful approach for aiding the interpretation of GWAS data and interrogating the relationship between CpG methylation and gene expression. Further development and application of this method may help improve understanding of the role of CpG methylation and gene expression in complex trait biology and to identify potential targets for disease therapy.

## AUTHOR CONTRIBUTIONS

Heather J. Cordell and Andrew P. Morris conceived and designed the project and played an important role in interpreting the results. James J. Fryett and Heather J. Cordell carried out data analysis and drafted the manuscript. All authors contributed to revising the manuscript and approved the final paper.

## CONFLICT OF INTEREST

The authors declare no conflict of interest.

## Supporting information

Supplementary information.

Supplementary information.

Supplementary information.

Supplementary information.

Supplementary information.

Supplementary information.

Supplementary information.

Supplementary information.

Supplementary information.

## Data Availability

ARIES (ALSPAC) and Understanding Society data are available by application to ALSPAC (http://www.bristol.ac.uk/alspac/researchers/access/) and Understanding Society (https://www.understandingsociety.ac.uk/documentation/health-assessment/accessing-data), respectively. Results generated during this study can be found within the published article and its supplementary files, with the final ARIES and Understanding Society methylation prediction models that we developed (.db files, ready for use with S‐PrediXcan) freely available for download from https://www.staff.ncl.ac.uk/heather.cordell/MethPaper.html.
