# Supplementary figures and images for: Investigating the prediction of CpG methylation levels from SNP genotype data to help elucidate relationships between methylation, gene expression and complex traits
Source: Genet Epidemiol. 2022 Aug 5;46(8):629–43. doi: 10.1002/gepi.22496 (PMC9804820; doi:10.1002/gepi.22496)

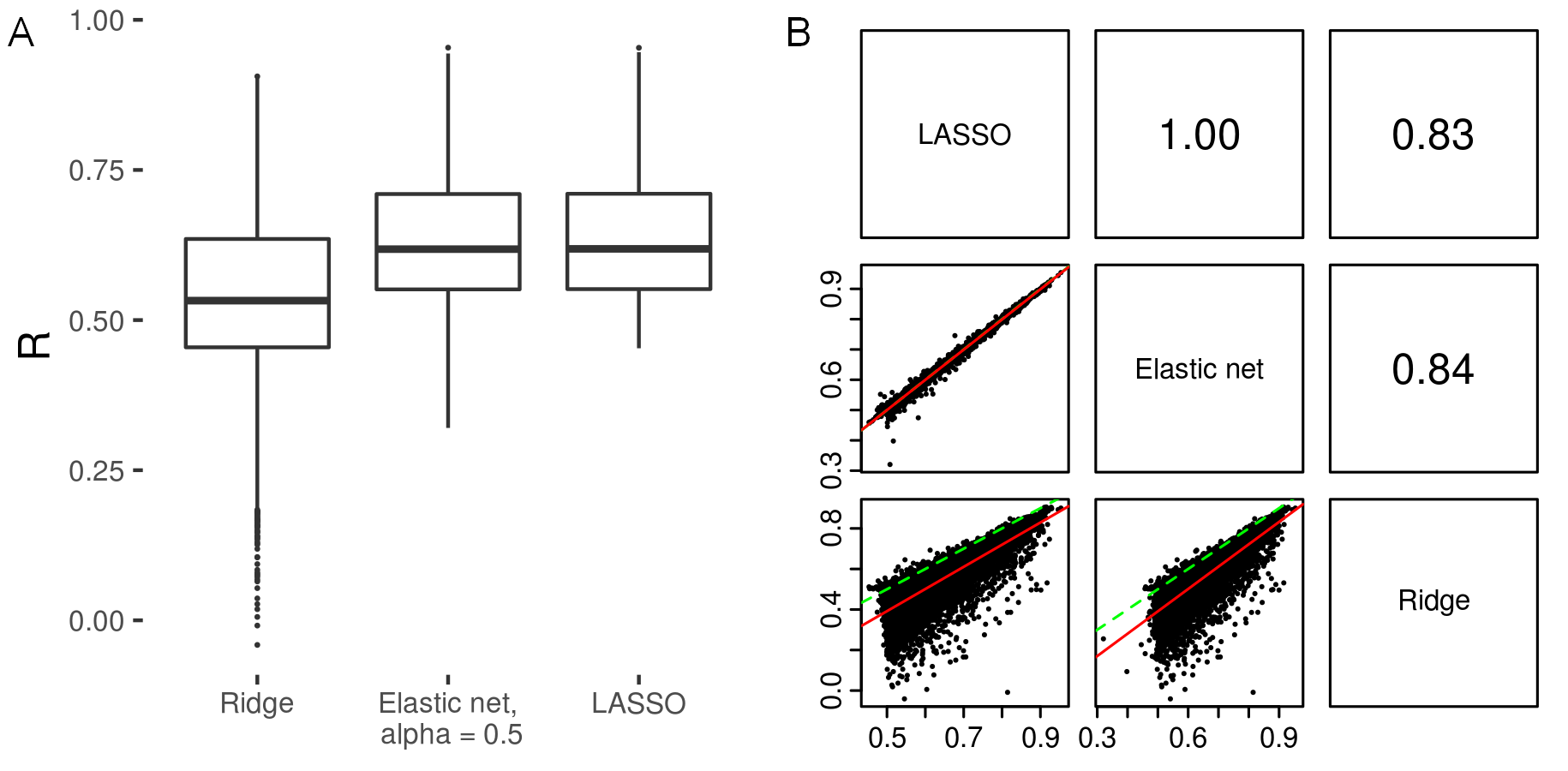

Supplement: Supplementary file 1 — Supplementary information. [file GEPI-46-629-s001.png]

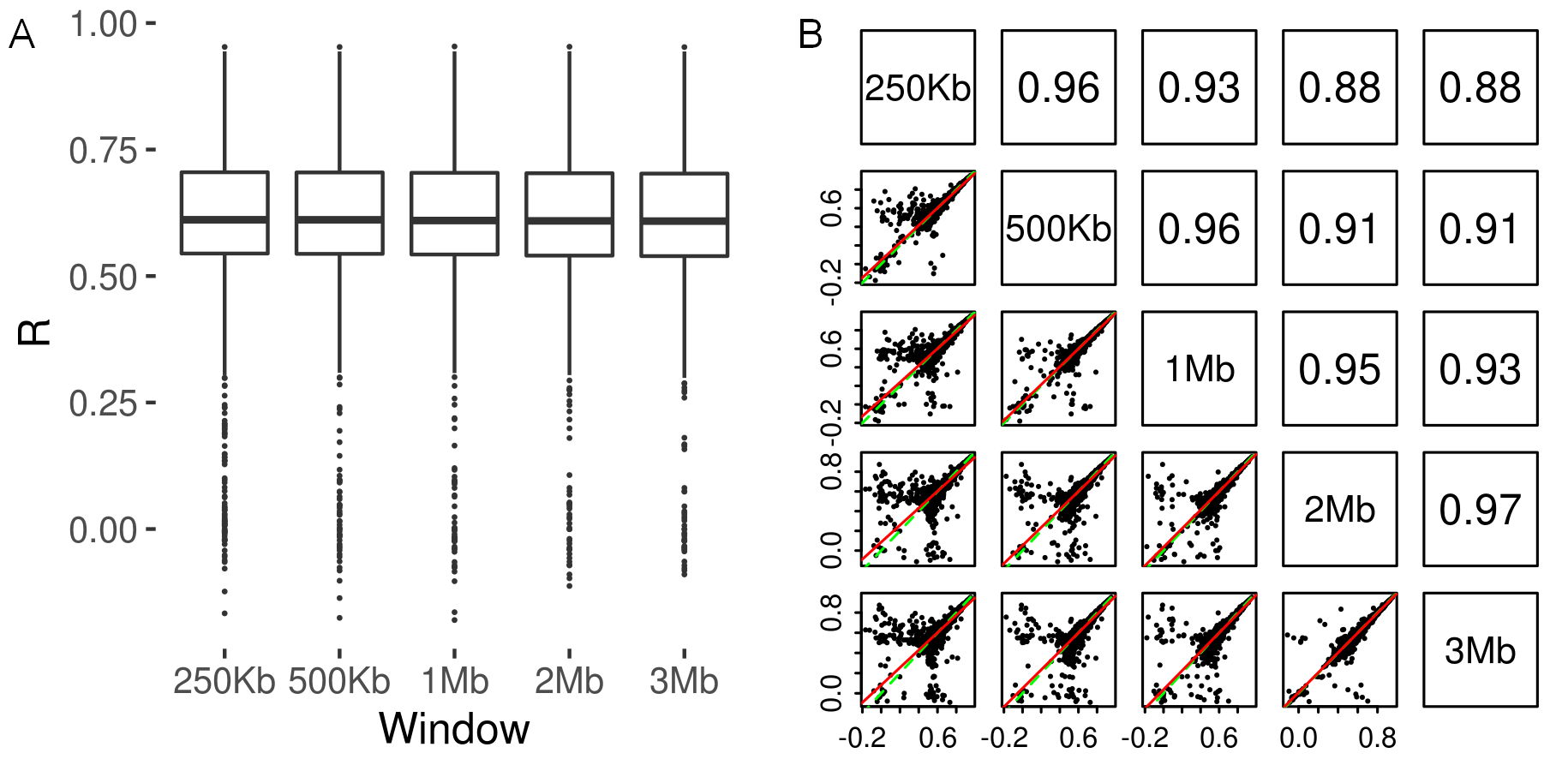

Supplement: Supplementary file 2 — Supplementary information. [file GEPI-46-629-s006.png]

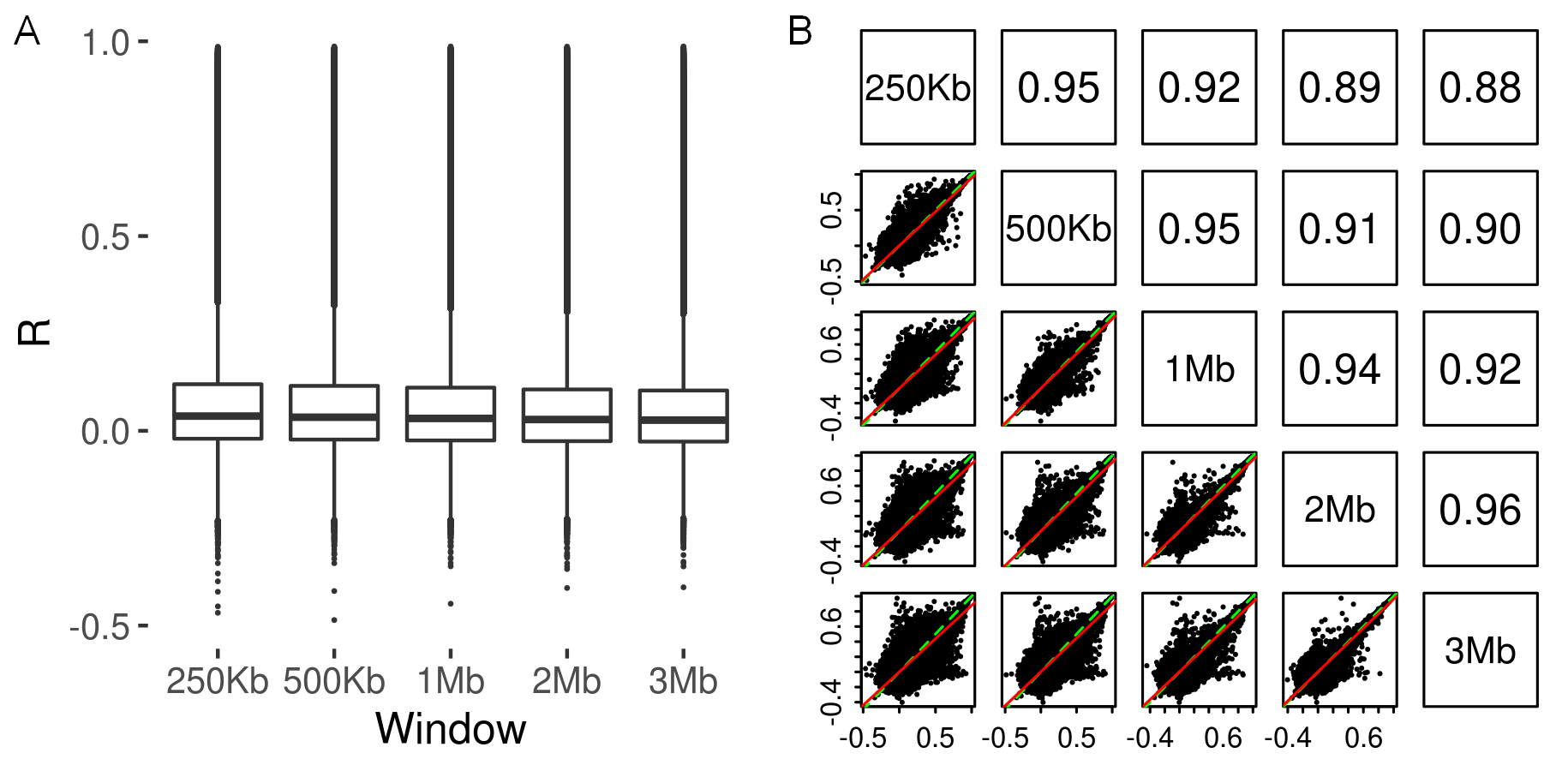

Supplement: Supplementary file 3 — Supplementary information. [file GEPI-46-629-s007.png]

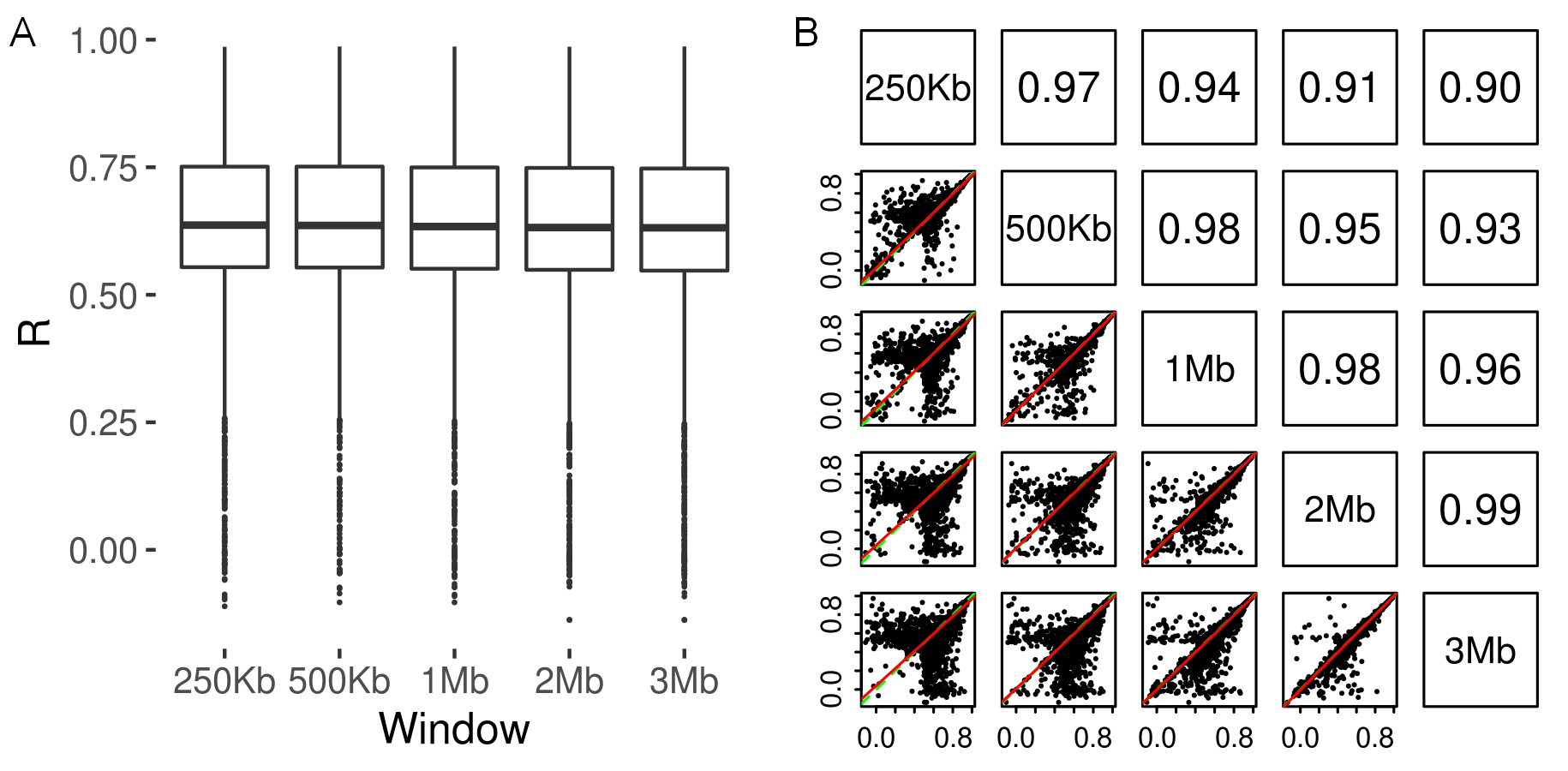

Supplement: Supplementary file 4 — Supplementary information. [file GEPI-46-629-s004.png]

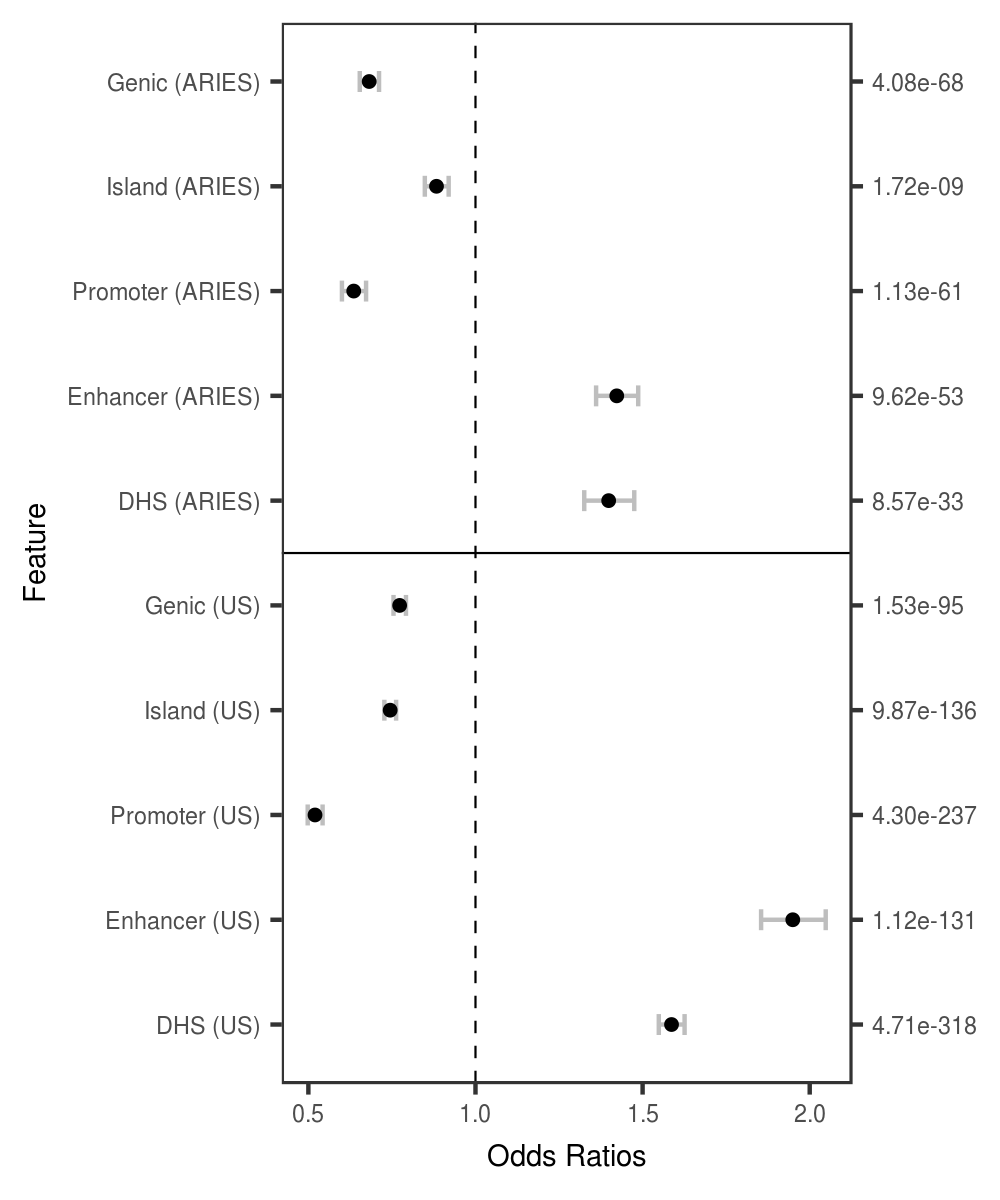

Supplement: Supplementary file 5 — Supplementary information. [file GEPI-46-629-s002.png]

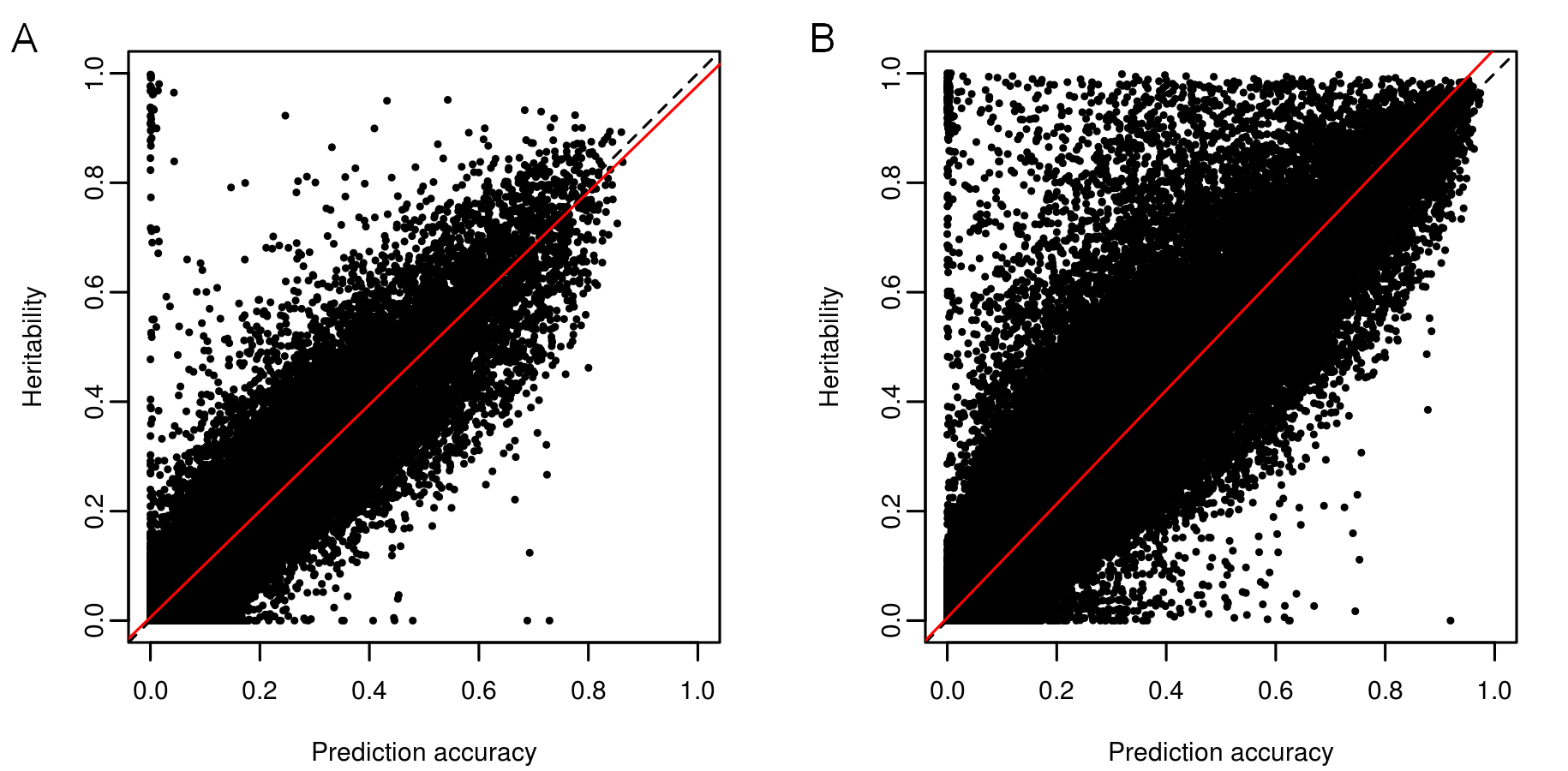

Supplement: Supplementary file 6 — Supplementary information. [file GEPI-46-629-s008.png]
