# Supplementary material for: Investigating the prediction of CpG methylation levels from SNP genotype data to help elucidate relationships between methylation, gene expression and complex traits
Source: Genet Epidemiol. 2022 Aug 5;46(8):629–43. doi: 10.1002/gepi.22496 (PMC9804820; doi:10.1002/gepi.22496)
Supplement: Supplementary file 9 — Supplementary information. [file GEPI-46-629-s009.docx]

**Supplementary figures and tables**

**Supplementary Figure S1. Comparison of penalised regression approaches for predicting CpG methylation for well predicted CpG sites.** Displayed are CpGs that achieved R estimates of 0.5 or greater from any of the 3 penalised regression approaches. (A) Box plots of prediction accuracy estimates (R) from training and testing prediction models using 3 forms of penalised regression (ridge regression, elastic net, LASSO) on ARIES data. The line within the box represents the median, with the edges of the box the upper and lower quartiles. (B) Correlation plots between prediction accuracy estimates achieved using the 3 penalised regression approaches. In the lower panels, each point represents a CpG site, with the R achieved by 2 methods displayed on the axes. Also shown are the line of equality (green dashed line) and a best fit line between x and y (red solid line). Upper panels show the pairwise correlations between the R values achieved using the 3 methods.

**Supplementary Figure S2. Comparison of window sizes for predicting CpG methylation for well-predicted CpG sites.** Displayed are CpGs that achieved R estimates of 0.5 or greater from any of the 5 window sizes. (A) Box plots of prediction accuracy estimates (R) from training and testing prediction models using 114 elastic net with SNPs selected using 5 window sizes (250Kb, 500Kb, 1Mb, 2Mb and 3Mb) on ARIES data. The line within the box represents the median, with the edges of the box the upper and lower quartiles. (B) Correlation plots between prediction accuracy estimates achieved using the 5 window sizes. In the lower panels, each point represents a CpG site, with the R achieved at the 2 window sizes displayed on the axes. Also shown are the line of equality (green dashed line) and a best fit line between x and y (red solid line). Upper panels show the pairwise correlations between the R values achieved at the 5 window sizes.

**Supplementary Figure S3. Comparison of window sizes for predicting CpG methylation using data from Understanding Society.** (A) Box plots of prediction accuracy estimates (R) from training and testing prediction models using elastic net with SNPs selected using 5 window sizes (250Kb, 500Kb, 1Mb, 2Mb and 3Mb) on ARIES data. The line within the box represents the median, with the edges of the box the upper and lower quartiles. (B) Correlation plots between prediction accuracy estimates achieved using the 5 window sizes. In the lower panels, each point represents a CpG site, with the R achieved at the 2 window sizes displayed on the axes. Also shown are the line of equality (green dashed line) and a best fit line between x and y (red solid line). Upper panels show the pairwise correlations between the R values achieved at the 5 window sizes.

**Supplementary Figure S4. Comparison of window sizes for predicting CpG methylation for well-predicted CpG sites using data from Understanding Society.** Displayed are CpGs that achieved R estimates of 0.5 or greater from any of the 5 window sizes. (A) Box plots of prediction accuracy estimates (R) from training and testing prediction models using elastic net with SNPs selected using 5 window sizes (250Kb, 500Kb, 1Mb, 2Mb and 3Mb) on ARIES data. The line within the box represents the median, with the edges of the box the upper and lower quartiles. (B) Correlation plots between prediction accuracy estimates achieved using the 5 window sizes. In the lower panels, each point represents a CpG site, with the R achieved at the 2 window sizes displayed on the axes. Also shown are the line of equality (green dashed line) and a best fit line between x and y (red solid line). Upper panels show the pairwise correlations between the R values achieved at the 5 window sizes.

**Supplementary Figure S5. Enrichment of CpG annotations among well-predicted CpGs tested in MWAS.** CpG sites were annotated using manifest files downloaded from the Illumina website. For each annotation, enrichment of well-predicted (R ≥ 0.5) CpGs against the background of all CpGs on either the 450k or EPIC chip was tested using a two-sided Fisher’s exact test. Odds ratios and 95% confidence intervals are shown on the x axis, with p values shown on the right.

**Supplementary Figure S6. Comparison of CpG methylation prediction accuracy estimates with estimates of the heritability of CpG methylation.** Box A shows the results from analysis using ARIES data, and Box B shows the results from analysis using Understanding Society data. In each box, each point represents a CpG site, with its prediction accuracy estimate obtained from training and testing a prediction model using the ARIES data shown on the x axis, and its heritability estimate obtained using GCTA REML analysis on the ARIES data shown on the y axis. The red line represents a best fit line, and the dashed line represents the line of equality (y=x). In this plot, prediction accuracy is shown as R^2^ rather than R, as the heritability is the upper bound on the estimate of R^2^.

**Supplementary Table S1:** Significant genes obtained when testing for association between PBC disease status and predicted gene expression.

**Supplementary Table S2:** Significant CpGs, genes and splice sites obtained when testing for association between PBC disease status and predicted methylation/gene expression/splicing. CpG results were obtained using Understanding Society models.
